# A web-based educational intervention to implement trauma-informed care in a paediatric healthcare setting: protocol for a feasibility study using pre-post mixed methods design

**DOI:** 10.1186/s40814-020-00636-8

**Published:** 2020-08-19

**Authors:** Megan Simons, Alexandra De Young, Steven M. McPhail, Gillian Harvey, Justin Kenardy, Sanjeewa Kularatna, Roy Kimble, Zephanie Tyack

**Affiliations:** 1 Occupational Therapy Department, Queensland Children’s Hospital, Children’s Health Queensland Hospital and Health Service, 501 Stanley Street, South Brisbane, Queensland 4101 Australia; 2 Centre for Children’s Burns and Trauma Research, Child Health Research Centre, The University of Queensland, 62 Graham Street, South Brisbane, Queensland 4101 Australia; 3 School of Psychology, The University of Queensland, St Lucia, Queensland 4072 Australia; 4 Australian Centre for Health Services Innovation (AusHSI) and Centre for Healthcare Transformation, School of Public Health and Social Work, Queensland University of Technology, 60 Musk Avenue, Kelvin Grove, Queensland 4059 Australia; 5 Clinical Informatics Directorate, Metro South Health, 199 Ipswich Road, Woolloongabba, Queensland 4102 Australia

**Keywords:** Implementation science, Paediatrics, Health knowledge, Training, Web-based, Trauma-informed care, Feasibility studies, Burns

## Abstract

**Background:**

Adoption of responsive trauma-informed practices by staff in hospital-based paediatric care may help mitigate downstream costs associated with treatment delivery due to reduced pain and distress for children and care providers, improved health-related quality of life and increased satisfaction with care. A web-based education intervention (termed Responsive CARE) was developed to build self-efficacy of staff in a paediatric medical setting. This protocol paper describes a feasibility study (including preliminary effectiveness) of the implementation of Responsive CARE in a tertiary, outpatient burn clinical setting.

**Methods:**

A pre-post, mixed methods design will be employed. Children and caregivers attending hospital for change of burn wound dressings or burn scar management during the 3-month control or 3-month intervention period will be eligible, with follow-up to 6-months post-baseline. All children and caregiver/s will receive “standard care” including burn interventions focused on wound healing, scar management, itch management (both pharmacological and non-pharmacological), counselling, age-appropriate procedural support and burn rehabilitation. Health professional participants will be those involved in the management of children with burns during the study period or their senior managers. Health professional participants who attend a weekly educational clinical meeting will be invited to complete the intervention during a 1-month timeframe between the control and intervention period (or upon their commencement in burn outpatients during the intervention period) using an individualised log-in process. A purposive sample of caregivers and health professionals will be sought for participation in semi-structured interviews. Qualitative data will be analysed using Framework analysis. Feasibility will be evaluated via interviews, digital records of intervention usage and technical assistance logs. The primary outcome measures of effectiveness (pain, itch and distress) will be measured using self-report or behavioural observation. Quantitative data will primarily be analysed descriptively and using generalised linear models.

**Discussion:**

This study will provide insights into factors that impact upon the feasibility of a web-based trauma-informed care education intervention in a clinical practice setting. This knowledge may support other education approaches within healthcare settings related to improving and supporting patients to reduce the risk of healthcare interactions that result in paediatric medical traumatic stress.

## Background

Hospitalisation for illness or injury can be experienced by up to 25% of children and their families/carers as traumatic or adverse life events [1]. A potentially traumatic event is considered to be any situation a child subjectively experiences as distressing or frightening [2]. In the medical context, the child’s reaction to traumatic events is defined as paediatric medical traumatic stress (PMTS). Up to 80% of hospitalised children and their caregivers experience at least one symptom of PMTS [3]. Around 30% will experience post-traumatic stress disorder (PTSD) within the first 6 months after injury, and 10% are at risk of experiencing a chronic PTSD symptom trajectory [1, 4]. PTSD in childhood has been associated with frequent psychiatric comorbidity [4], increased pain [5], poorer treatment adherence [6], diminished health-related quality of life [1, 3] and functional impairment at similar levels to those experiencing chronic illnesses (e.g. diabetes and cancer) compared to injury [7, 8].

Trauma-informed care requires recognition and understanding of the widespread impact of trauma and recognising how it presents differently across the lifespan. It is also means being responsive and applying trauma-informed care knowledge into practice to minimise the risk of further traumatisation [9, 10]. Adoption of sound trauma-informed care practices in hospital-based paediatric care may help mitigate the downstream costs associated with treatment delivery [11]. For example, a small to medium reduction in PTSD symptom severity scores in children and a medium to large reduction in arousal symptoms in young children were demonstrated following early and aggressive treatment of pain following burn injury [12, 13]. In another study, when children were screened as at ‘high’ risk for PTSD, the implementation of a 2-session early intervention program designed to prevent persistent trauma reactions in young children (aged 1 to 6 years) showed a moderate reduction in PTSD symptom severity scores during the first 3 months post-injury [14]. Integrating psychosocial with physical care can reduce psychological distress, physical symptoms and pain, improve quality of life, coping and treatment adherence [8, 15] and ultimately reduce the burden and costs of healthcare utilisation [16, 17].

Whilst most healthcare staff recognise the need for trauma-informed care, few receive training that facilitates consistency in practice and implementation [18, 19]. An international survey (*N* = 2648) identified substantial knowledge gaps among hospital-based health professionals regarding child traumatic stress and trauma-informed care [20]. These results were reflected in a staff survey (*N* = 180) at a tertiary paediatric hospital setting in which approximately 60% of knowledge questions about child traumatic stress and trauma-informed care were answered correctly [21]. In a companion study undertaken at the same centre using observational methods to record the incidence of trauma-informed care practices, the frequency of using trauma-informed care practices was low, varying from 0.07% (use of screening tools) to 29% (asking about needs/concerns) [22]. Most staff reported they rely on visible triggers of distress or need before providing trauma-informed care [21]. The authors concluded that training that improved confidence and knowledge of trauma-informed care practices may change staff behaviours and impact on staff well-being [22].

**Fig. 1:**
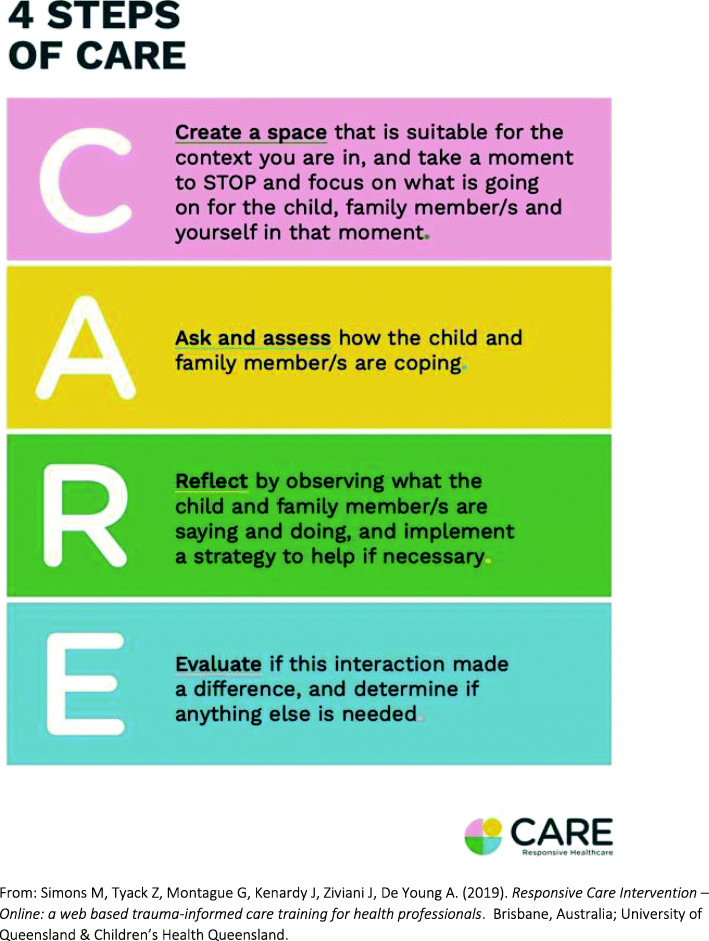
The Responsive CARE Framework [25]

It is generally considered that web-based courses allow for iterative knowledge acquisition and application in real-world practice settings, at a relatively low cost [23, 24]. In a paediatric hospital setting, Moss and colleagues also found that training indirectly predicted the application of trauma-informed care via improved knowledge and self-efficacy, suggesting that a training process that promotes improved knowledge and confidence is more likely to be effective [22]. In that study, staff expressed a preference for online training modalities. Following this, some of the authors of the current study (MS, ADY, ZT, JK) developed a fully automated, interactive web-based staff training intervention (herein termed Responsive CARE) in collaboration with a panel of seven national and international experts (see acknowledgements) in trauma-informed care practices and adult education (for content development and usability testing). The purpose of the evidence-informed intervention was to give clinicians a way to easily remember and integrate the D-E-F protocol (https://www.healthcaretoolbox.org/) into everyday clinical interactions with patients, family members and hospital staff based on the Responsive CARE Framework (Fig. 1) [25]. We coined the term ‘responsive trauma-informed care’ to describe healthcare delivery that incorporates an understanding of the impact of stress on clinical care with ill or injured children; is underpinned by trustworthiness, choice, collaboration and empowerment; and is responsive to the impact of trauma on the physical, psychological and emotional well-being of children, their families and staff. However, whilst it is acknowledged that trauma-informed care is likely beneficial, evidence of the effectiveness of training methods on changes to clinician behaviour and long-term patient outcomes is still limited [26].

The Kirkpatrick Model has previously informed the evaluation of web-based training programs for healthcare providers [27, 28]. The New World Kirkpatrick Model is an updated evaluation framework comprising of four domains (reaction, learning, behaviour, results) in addition to affective elements such as the participant’s perceptions of relevance of course content, engagement with learning activities and attitude [29, 30]. *Reaction* includes the evaluation by a participant to the instructor, setting, material and learning activities. It is considered that higher levels of satisfaction, whilst not guaranteeing learning, are more likely to increase the probability of it occurring [31]. *Learning* involves determining the extent to which learning has occurred and may include activities such as case scenarios or a pre- and posttest situation to demonstrate learning [31]. Behaviour seeks to determine the extent to which new skills and knowledge have been applied, as even with high levels of satisfaction and the achievement of learning objectives, it must not be assumed that the transfer of knowledge into behaviour will occur [31]. This framework [29] will be used as a basis for the current study to consider the presence of four conditions considered essential for a change in behaviours to occur: (1) a desire for change to occur, (2) to know what to do and how to do it, (3) work in the right climate (i.e. the work environment must be receptive to the transfer of the learning) and (4) be rewarded for changing. These rewards may be either intrinsic to the individual (such as the satisfaction of doing a job well) or extrinsic (economic) in their nature [27]. Interviewing those impacted by the behaviours of the individual (e.g. patient impacted by clinician behaviours) is a means of evaluating the extent to which new skills and knowledge have been applied [31]. The evaluation of results involves an analysis of system-wide or organizational impact which extends beyond the scope of most training course evaluations.

In regard to trauma-informed care, evaluation of web-based educational programs has considered reaction and learning evaluation to date. When the efficacy and acceptability of a 15-min web-based training program on PMTS and trauma-informed care were examined using parallel group superiority randomized controlled trial, paediatric emergency department staff (nurse or physician) demonstrated greater knowledge following training and at follow-up 1-month later than the control group (*F* = 41.66, *p* > .001) [32]. However, a systematic review of studies that evaluated the effects of organizational interventions that included a trauma-informed staff education component has concluded that whilst staff knowledge, attitudes and self-reported behaviour related towards trauma-informed practices improved immediately after participating in training, a sustained effect was less clear [26]. Trauma-informed interventions that appeared to have the most impact on patient outcomes were those in which other components such as organizational policy changes occurred alongside training. This finding suggests that the implementation processes that result in the embedding of trauma-informed care practice as routine are complex and highlight the likely importance of different organizational levels as part of implementation strategy [19]. However, no web-based interventions were included in the systematic review [26]. Whilst randomised trials of homogenous populations are considered the gold standard for evaluating the efficacy of interventions, these study designs have drawbacks including being expensive to conduct and may be perceived as irrelevant to the more complex study setting, making implementation of the study findings challenging [33]. This study aims to the following: (1) explore the feasibility of the web-based Responsive CARE intervention on self-efficacy of health professionals to implement trauma-informed care in a paediatric healthcare setting and (2) explore the short-term preliminary effect of delivering this intervention. This information will be used to adapt the Responsive CARE intervention (or develop multi-component interventions) and implementation strategy for the delivery of responsive trauma-informed care in a paediatric healthcare setting.

The specific objectives for the feasibility study [34] are to determine the following:

1. The acceptability of the Responsive CARE intervention to health professionals
2. The demand for trauma-informed care training and use (or intended use) of the Responsive CARE intervention by health professionals in a tertiary, outpatient burn clinical setting
3. To what extent the Responsive CARE intervention training can be delivered using existing resources and infrastructure within the tertiary, clinical setting
4. Barriers and enablers in the implementation environment and feasibility of the recruitment pathway to inform the amount and type of resources at different organizational levels (e.g. clinician, supervisor, manager) required to implement the intervention successfully

Specific objectives for the investigation of patient-centred outcomes are to:

1. Determine preliminary evidence of effectiveness of the intervention on a health professional’s knowledge, attitude and behaviours. This will provide preliminary indication of the extent to which skills and knowledge following the completion of the intervention have been applied and an evaluation of factors that drive clinician behaviour change.
2. Determine preliminary evidence of effectiveness of the intervention on child’s acute pain, itch, distress (i.e. traumatic stress reactions), caregiver’s treatment satisfaction and health-related quality of life. This will provide an indication of whether the intervention can show change within this group. It is hypothesised that child participants will experience less acute pain, itch and distress in the posttest compared to the pretest period, and caregiver participants will experience enhanced treatment satisfaction in the posttest compared to the pretest, following staff completion of the responsive CARE intervention.
3. Estimate healthcare resource use and costs of delivering the intervention to inform cost-related modelling for use in future economic evaluations of web-based education platforms.

The TIDierR checklist [35] and CONSORT-EHEALTH checklist (V.1.6.1) [36] have been used to detail the intervention and evaluation and CONSORT extension to pilot and feasibility trials [37] for reporting the study protocol.

## Method

### Study design

The feasibility study will employ a pretest posttest observational design using mixed methods to evaluate acceptability, demand, implementation and practicality [34] of the Responsive CARE intervention. Using a mixed methods approach will allow for the collection of quantitative data in terms of the demand for the intervention as well as rich qualitative data to understand the factors affecting implementation that may ultimately impact on the intervention’s sustainability and/or scalability. Purposive sampling will be used for the qualitative study components. Interviews will cover five main areas: understanding of trauma-informed care practice, experience of trauma-informed care, clinician’s readiness to change, acceptability of the Responsive CARE intervention and identify potential barriers and enablers to implementing an e-learning package within organizational culture.

A mixed method, pretest posttest comparison design will be used to examine preliminary effectiveness, with consecutive sampling of children and caregivers. Baseline quantitative data will be collected during a 3-month control period followed by a 3-month intervention period. Follow-up of participants will be completed at 3 months and 6 months after baseline for participants in the control and intervention periods. Between the control and intervention periods, there will be a 1-month period to implement the e-learning intervention. Qualitative interview data will also be collected from caregivers in the control and intervention periods, who will be purposively sampled.

### Participants

#### Inclusion criteria

All children and caregivers attending an outpatient service for change of burn wound dressing or burn scar management during the control or intervention period will be eligible to participate. Health professional participants (e.g. medical staff, occupational therapists, nursing staff, social workers, senior managers) will be those involved in the management of children with burns during the study period. Students who are members of the treating burn team for less than 2 weeks during the study will be excluded.

#### Exclusion criteria

Exclusion criteria are no caregiver or legal guardian who is able to provide informed consent, caregivers who are unable to speak or to understand English unless an interpreter is present and children attending the burn outpatient service with a condition other than a burn injury.

### Study setting

The study will be conducted at the outpatient clinic for children with burns at a specialist publicly funded, teaching children’s hospital located in Brisbane, Australia. The clinic accepts referrals for all children and young people with burns (0–18 years) across the state of Queensland and Northern New South Wales and receives approximately 1300 new referrals per year. The burn clinic team is a multidisciplinary team comprised medical team (5 burn consultants and four teams of rotational registrars and residents), specialist burn nursing staff (1 burn clinical nurse consultant, 1 burn clinical nurse and up to 3 registered nurses), allied health staff (1–2 physiotherapists, dietitian, social worker, music therapist, consultant liaison mental health service and up to 6 occupational therapists), as well as up to 4 administration and 5 higher degree research staff. Core staff in attendance at each burn outpatient clinic include medical, nursing, allied health (allied health assistant, occupational therapy, physiotherapy, social work) and administrative staff. Burn specific experience ranges from < 1 month to over 30 years within the multidisciplinary team. There are generally up to 2 clinical trials or other research projects occurring within the recruiting centre at any time of the year. At the time of the study, the organizational approach towards educational or care strategies to reduce paediatric medical traumatic stress in burn outpatients was referral to psychosocial services (generally social work) and participation in a clinical trial (if aged below 6 years and screened at high risk for PTSD) that commenced at the beginning of this study [14].

### Recruitment

#### Quantitative study component

The sample size will be based on children available to be recruited during the control and intervention period, which is anticipated to be 180 children (based on an average of 30 new referrals each month to the burn centre). It is anticipated up to 50 health professionals may be eligible to access the intervention, with an estimated 50% turnover of staff in the training and intervention period (as the recruiting centre is a teaching hospital). Health professionals that commence working at the recruiting centre during the intervention period will be informed about the study by a clinician researcher (MS) or research assistant and offered personal log-in details following informed consent.

#### Qualitative study component

Semi-structured interviews or focus groups (for health professionals only) will be conducted with approximately 10 parents and 10 multidisciplinary burn team members involved in the care of patients across the control and intervention periods. Up to two interviews per participant may be conducted. The researchers will recruit a purposive sample with participants including senior clinical leaders, members of each professional group (medical, allied health, nursing) and clinicians of variable levels of experience. Semi-structured individual interviews will also be conducted with up to 5 senior managers (who are line managers for multidisciplinary burn team members) during the study period.

### The Responsive CARE intervention—the intervention

The fully automated web-based Responsive CARE intervention consists of four modules (see Table 1), ideally completed sequentially. The intervention package includes a pre- and post-course quiz to self-assess knowledge and allows users to track their progress (see Additional File 1). Interactive elements such as case scenarios allow the user to receive immediate feedback and respond accordingly. Upon completion of the final module, the user is asked to provide course feedback using an automated form (see Additional File 2). Upon completion of all modules, quizzes and survey, the user is able to access an automated certificate of completion.

**Table 1:** The modules and their content for the Responsive CARE intervention

Module	Description
1. Understanding paediatric medical traumatic stress	This module explores what is traumatic about medical experiences and what influences an individual’s response to the same experience. It describes typical responses to PMTS and patterns of recovery, including when specialist help is indicated.
2. Introducing the CARE Framework	All healthcare professionals can identify, prevent or minimise the impact of paediatric medical traumatic stress reactions on ill or injured children, their families and themselves. This can be achieved by integrating responsive trauma-informed care into their standard practice throughout all stages of medical care. This module explores how to use responsive trauma-informed care in practice using the evidence-based CARE framework.
3. General skills for applying CARE	This module explores how health professionals can provide responsive trauma-informed CARE. Health professionals are informed about how CARE was designed including using flexible delivery methods and so as to not add additional work or responsibility to a health professional’s routine. Areas of discussion include the following: managing disclosure and transitions in a trauma-informed manner, the importance of reflective practice when considering scope of practice, and how health professionals can provide responsive trauma-informed care even when time is limited. Cultural considerations for responsive trauma-informed care, as well as considerations for children with special education needs are addressed.
4. Taking CARE of yourself	This module explores the challenges that may arise for health professionals working with children and families who experience medical traumatic stress. Checklists to increase self-awareness of signs of stress in this setting, as well as evidence-based strategies to manage their own well-being are provided.

The Responsive CARE intervention will be freely available to all health professionals working in burn outpatients via ilearn.health.qld.gov.au from the commencement of the 1-month training period (June 2019) and until the 6-month follow-up intervention period is completed (April 2020). Technical assistance is available via web-based or hotline numbers provided on the ilearn site. No changes will be instigated to the content of the Responsive CARE intervention throughout the intervention period (including 6-month follow-up). No automated prompting will be provided to the user once enrolled. New health professionals to the burns centre during the intervention period and 6-month follow-up will be informed of the study and undergo training of how to access the site following informed consent with clinician researcher (MS) or research assistant as close to commencing in the recruiting centre as possible. It is anticipated that open access to the responsive CARE intervention will be available from ilearn.health.qld.gov.au at the completion of data collection. Testor accounts are currently available upon request to the author (MS). Alternatively, an overview of the content is provided in Additional File 3.

**Fig. 2:**
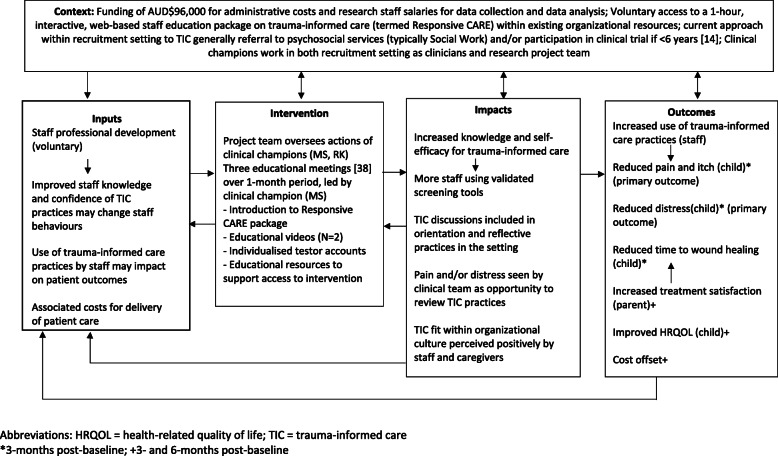
Logic model for evaluation of Responsive CARE intervention

A logic model has been developed (see Fig. 2) to demonstrate the predicted outcomes and impact of the intervention.

### Procedures

**Fig. 3:**
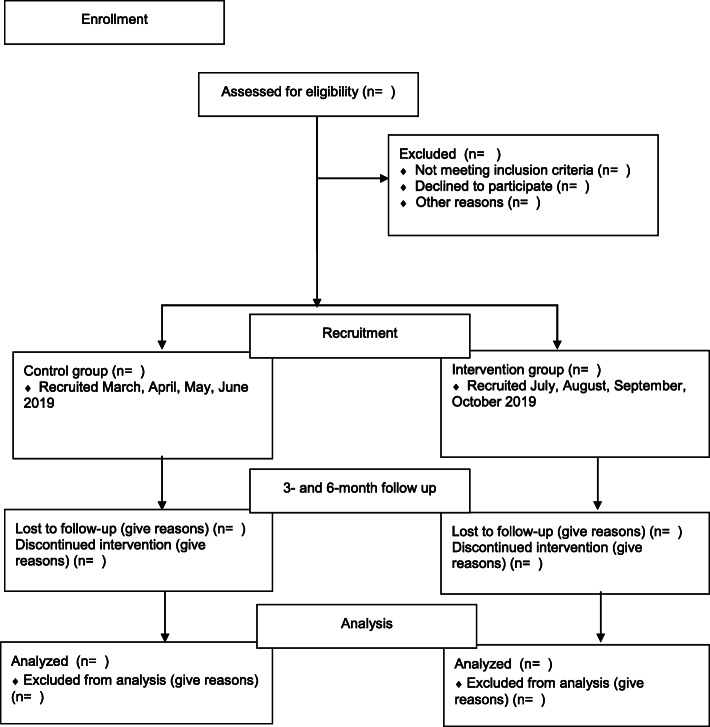
Flow diagram of enrolment in feasibility study

Eligible child and caregiver participants who attend during the study period will be approached by a research assistant at their outpatient burn clinic appointment and informed of the study and invited to participate in quantitative data collection (see Fig. 3). Data will be collected using iPads (or pencil and paper if preferred by the participant) in the outpatient clinic setting. Interviews will be completed with caregivers on-site at the recruiting centre or via the telephone (if preferred) when convenient for the participant. Interviews will be audio-recorded and transcribed verbatim.

Health professional participants who attend an existing weekly educational clinical meeting at the recruiting centre during week 1 (of the 1-month training period between the control and intervention period) will be invited to complete the Responsive CARE intervention. In week 1, a 10-min educational demonstration (Additional File 4) of how to access the web-based intervention, as well as an overview of the content will be delivered in a burn educational meeting by champions (MS, RK). Personal log-in details for health professionals (that are not connected to staff records) will be provided following informed consent regarding details for accessing the web-based intervention (Additional File 5). Staff are able to access the intervention from any web-based platform of their choosing. The training will be repeated weekly (if desired by the clinical team) or individually upon request throughout the 1-month training period. Educational demonstrations in week 3 will also include an interactive whiteboard video (6 min) on responsive trauma-informed CARE and a 7 min interactive whiteboard video on self-CARE in week 4. Researchers will record the allocated log-in details so they are available upon request by any user. Verbal reminders of the invitation to participate will be repeated at the weekly educational clinical meeting throughout the 1-month period. Health professionals will be able to access the intervention using their personal log-in until completion of the intervention period follow-up data collection (i.e. 6-months post-baseline). Upon commencing the responsive CARE intervention, health professionals will complete a survey to consent (or not) to research and complete demographic details such as their profession, gender, age, years of experience with hospital-based care, years in current burns role and previous training in trauma-informed care (see Additional File 6).

All health professionals working within the burn clinical team at the time of recruitment will be informed at the weekly educational clinical team meeting or approached individually by a researcher and invited to participate in a 1:1 interview or focus group on-site at the recruiting centre. Senior managers will be approached individually by a senior researcher and invited to participate in a 1:1 interview. Interviews and focus groups will be audio-recorded and transcribed verbatim. Transcripts will be returned to health professionals for member checking.

### Implementation plan

The Consolidated Framework for Implementation Research (CFIR) [38] will be used to guide evaluation of the factors influencing implementation identified through qualitative interviews and technical assistance logs, knowing these factors will improve understanding of the barriers and enablers to implementation of the intervention as planned and proposed. The CFIR (a widely cited and rigorously developed determinants framework for implementation) consists of five domains, with each domain consisting of a number of constructs identified in the literature as impacting successful implementation. These findings will be analysed and actioned to inform adaptations and future implementation blueprint.

### Outcomes

#### Feasibility outcomes

Acceptability (level of satisfaction, intention to continue use and perceived appropriateness of the intervention) and practicality (perceived sustainability within existing infrastructure) of the intervention, as well as factors affecting implementation will be explored through the qualitative interviews with staff carried out throughout the study period. Actual use (versus expressed interest or intent to use) will be observed via ilearn digital records to assess demand for the intervention. Signs of distress on patient-centred outcome measures (see below) during the control period will also be used to assess the need for trauma-informed care. An attrition diagram will be developed demonstrating usage, dose and engagement. Implementation outcomes will explore the perceived impact of the responsive CARE intervention on clinician behaviours (for example, use of validated screening tools, empathic communication strategies) according to quantitative and qualitative data collected from staff and caregivers, as well as barriers and enablers in the implementation environment. Changes required to support the uptake of the Responsive CARE intervention in the training and intervention period will be recorded in technical assistance logs in which the ongoing consultation process, any top-up training and alterations to processes will be recorded using narrative description.

### Primary patient-centred outcomes

The primary outcome measures of effectiveness to be tested for the main study are pain (self-report or behavioural observation), itch and distress. The Face, Legs, Arms, Cry, Consolability (FLACC) pain scale [39, 40] will be completed by health professionals as a behavioural measure of pain severity. An 11-point numeric rating scale (NRS) will be used by parents and children 8 years or older to rate pain and itch intensity [41]. The NRS is scored from zero to ten, with zero equal to “no pain or itch” and ten equal to “unbearable pain or itch”. Distress will be measured using the Paediatric Emotional Distress Scale—Early Screener [40, 42] for children below 6 years and the Child Traumatic Stress Questionnaire [43] for children aged 7 years and above.

Patient-centred outcome measures to be collected at baseline, 3-months and 6-months post-baseline are summarised in Table 2.

**Table 2:** Patient-centred outcome measures and timepoints when administered

Area of focus	Completed by			Outcome measure	Study period (control and intervention)		
	HP	C	YP		Baseline	3-months	6-months
Pain and itch (primary outcome)	X			The Face, Legs, Arms, Cry, Consolability (FLACC) [39, 40]	X	X	
		X	X	Numeric Rating Scale [41]	X	X	
Distress (primary outcome)		X		Pediatric Emotional Distress Scale – Early Screener^a^ [42]	X	X	X
			X	Child Traumatic Stress Questionnaire^b^ [43]	X	X	X
Stress		X		Perceived Stress Scale [44]	X	X	X
Satisfaction with treatment		X	X	Numeric Rating Scale (0 to 10)	X	X	X
Health-related quality of life		X	X	Brisbane Burn Scar Impact Profile [45–47]		X	X
		X	X	EuroQol-5D-Y^c^ [48, 49]	X	X	X
		X	X	CHU-9D^d^ [49, 50]	X	X	X

*HP* health professional, *C* caregiver, *YP* young person aged over 8 years (unless otherwise indicated)

^a^ For children below 6 years

^b^ For children aged 7 years and older ^c^ Administered to caregivers of children 3 years and older  ^d^Administered to caregivers of children 2 years and older

### Primary health professional outcomes

Change in knowledge scores and self-efficacy will be measured using questions designed for the study and recorded as part of the online package (see Additional File 1 and 2).

Secondary and healthcare resource outcomes to be collected at baseline and 3-months post-baseline in the control and intervention period are as follows:

1. Administered pain relief: the number (percentage) of patients administered first and second line medications will be recorded. The dose of pain relief and other medication for symptom management will also be recorded.
2. Rate and adjunct interventions (e.g. music therapy, counselling) for pain relief or reduction of stress symptoms.
3. Number of psychosocial or mental health referrals: this will be recorded from medical records.
4. Patient attendance rates at booked appointments and patient reminders: the percentage of booked appointments attended will be measured as well as the number of patient reminders sent by text, mail or phone as per standard practice.
5. Time to wound healing: this will be measured using the time taken in days from the burn injury to ≥ 95% of the wound area re-epithelialized with no further wound dressings required. Re-epithelialization will be assessed by experienced burn consultant or specialised burn nursing staff.

In addition, hospitalisation-related resource use and costs will be collected from hospital administrative records for 12-months prior to the control period for each patient and for the 6-months after the intervention period for each patient for informing cost-related modelling for use in future economic evaluations of web-based education platforms.

Sociodemographic and clinical data will be collected from the following:

(1) The Queensland Paediatric Burns Registry or medical records (maintained by the Centre for Children’s Burns and Trauma Research) including percent total body surface area (TBSA), percent full thickness burn, length of time post-burn, spontaneous skin healing versus grafted site, type of burn and length of time to re-epithelialisation.

(2) Patient and/or caregiver self-report using a standard sociodemographics and clinical data form and questionnaires collecting data on household education, employment, income and occupation, scar location, skin type, age, gender and comorbidities

(3) Fidelity of the intervention based on the commencement and completion rate

(4) Intervention details: time taken to complete the intervention, number of times accessed and number of staff who complete the package per discipline

These other outcomes will be used to describe the control and intervention groups and use of the intervention, as well as to be entered into analyses as predictors or confounding factors to be controlled for.

### Data analysis

#### Feasibility

Health professional and participant interviews and a technical assistance log will be independently coded using Framework analysis [51] based on CFIR domains and subsequently discussed by the two coders. This analysis will determine the acceptability and practicality of the Responsive CARE intervention and identify key contextual factors that impacted upon implementation. A third coder will be consulted if required to achieve consensus. From this, a network of core themes and sub-themes will be developed, informed by the CFIR constructs to guide evaluation of the factors that influenced implementation. Results from audits of ilearn digital metrics will inform assessment of demand and response to implementation strategies [52]. The descriptive data from the technical assistance logs will be analysed at project completion to quantify the number and type of issues experienced in implementation and the modifications (planned or unplanned) to the implementation blueprint. This data will be compared with ilearn digital records (of demand) to determine the overall feasibility of the Responsive CARE intervention in the burns outpatient clinic.

#### Patient-centred outcomes

Sociodemographic, clinical and intervention data will be summarised using descriptive statistics (e.g. mean and standard deviation and 95% confidence intervals) and presented for each group separately. Potential confounding variables for the outcomes are considered a priori to be the following: days to re-epithelialisation, percentage maximum burn depth, days post-burn at baseline, type of injury and number of surgical procedures (e.g. skin grafting) and severity of scarring and child traumatic stress symptoms. Potential confounding variables that are identified as significantly different or different to a clinically meaningful extent between the control and intervention group will be controlled for in analyses where appropriate. As this is a feasibility study, the study will likely be underpowered; therefore, quantitative outcomes will be interpreted only as preliminary findings.

Control period participants will be matched with intervention period participants on factors including age, gender, education and total body surface area burned to examine between group differences. Differences in outcomes between the groups will be analysed using generalised linear (mixed) models or equivalent analyses to examine differences at 3-month and 6-months post-procedure or 3-months and 6-months post-burn where applicable, adjusting for the baseline value of the outcomes where appropriate (with terms included for stratification and confounding variables where applicable). A sensitivity analysis will be conducted using imputation techniques to replace non-ignorable data that is considered to be missing at random over the follow-up period, to determine whether bias is likely in the complete case analysis.

Patient-centred outcome data will be stratified by age to coincide with different versions of patient-reported outcome measures for different age groups and caregiver report versus child report.

The mean difference in change scores for health professional’s knowledge and self-efficacy will be calculated for individual questions and analysed using a Wilcoxon paired test (assuming parametric assumptions are not likely to be observable in this relatively small sample).

### Data storage

Information collected as part of this study will be stored in a locked filing cabinet and in password-protected computer files. Any re-identifiable information obtained in this study will remain confidential and securely stored for 5 years as recommended by the National Health and Medical Research Council Guidelines (NHMRC).

## Discussion

Research is needed to evaluate whether web-based educational interventions focused on trauma-informed care practices can be feasibly used in clinical practices, potentially impact on patient outcomes and be cost-effective within real clinical contexts [53]. Through the use of an implementation science theory and logic model of evaluation, the process of translating web-based educational packages that aim to improve clinical care can be more thoroughly examined. Barriers and enablers within the healthcare system or implementation approach can be identified to better understand the uptake of this intervention into routine practice [54].

## Limitations

The use of a pretest posttest study design for feasibility testing of the intervention has limitations, in that pretest posttest studies do not have control over things that might be changing at the same time as the intervention, so outcomes cannot be attributed to the intervention alone [55]. However, in health services research, these study designs can be valuable to inform a more holistic, population-based, ecological systems science that attends to contextual factors and external validity [56].

## Future directions

Identifying which factors are important for successful implementation can support healthcare settings to consider the appropriateness of educational interventions in their setting. It is anticipated that the study findings will inform educational providers in a healthcare context regarding factors to consider in the process of improving and standardising education approaches across a range of clinical care specialist areas.

## Conclusion

A pre-post, mixed methods design will be employed to examine the feasibility and short-term effect, as well as resource use and costs, of a web-based trauma-informed care educational intervention for staff in a tertiary hospital, outpatient clinical practice setting. It is anticipated that use of implementation science theory and logic model of evaluation will provide insights into factors that impact upon the feasibility of the intervention in a clinical practice setting. This knowledge may support other education approaches within healthcare settings related to improving and supporting patients to reduce the risk of healthcare interactions that result in paediatric medical traumatic stress.

## Supplementary information

**Additional file 1.** Knowledge questions. Description: Questions to assess knowledge pre and post Responsive CARE intervention

**Additional file 2.** Feedback questions. Description: Questions to assess confidence, satisfaction and beliefs post Responsive CARE intervention

**Additional file 3:.** Overview of Responsive CARE course content. Description: Additional information about the Responsive CARE course content

**Additional file 4.** Introduction to Responsive CARE. Description: Slide content of introduction session for recruitment of users of the Responsive CARE intervention.

**Additional file 5.** Educational material for staff to support access to Responsive CARE intervention

**Additional file 6.** Demographic questions for Health Professionals. Description: Additional questions to describe health professionals completing Responsive CARE intervention

## Data Availability

The datasets used and/or analysed during the current study are available from the corresponding author on reasonable request if appropriate permissions are obtained (by those seeking to access the data) from the data custodians with appropriate ethical and governance approvals from Children’s Health Queensland Hospital and Health Service Human Research Ethics Committee who can be contacted at the following email: CHQETHICS@health.qld.gov.au. The author MS can be contacted at Megan.Simons@health.qld.gov.au for further information regarding access to the dataset.

## Abbreviations

CFIR: Consolidated Framework for Implementation Research
CHU: Child Health Utility
CONSORT: Consolidated Standards of Reporting Trials
FLACC: Face, Legs, Arms, Cry, Consolability
HRQOL: Health-related quality of life
NHMRC: National Health and Medical Research Council
NRS: Numeric rating scale
PMTS: Paediatric medical traumatic stress
PTSD: Post-traumatic stress disorder
QALY: Quality-adjusted life year
SPPBC: Stuart Pegg Paediatric Burns Centre
TBSA: Total body surface area
TIC: Trauma-informed care
TIDieR: Template for Intervention Description and Replication
