# Supplementary material for: A web-based educational intervention to implement trauma-informed care in a paediatric healthcare setting: protocol for a feasibility study using pre-post mixed methods design
Source: Pilot Feasibility Stud. 2020 Aug 19;6:118. doi: 10.1186/s40814-020-00636-8 (PMC7436985; doi:10.1186/s40814-020-00636-8)
Supplement: Supplementary file 1 — Additional file 1. Knowledge questions. Description: Questions to assess knowledge pre and post Responsive CARE intervention [file 40814_2020_636_MOESM1_ESM.doc]

| Number | Question | When completed? | |
| --- | --- | --- | --- |
|  |  | Prior to e-course | After  e-course |
| Introduction | Please complete the following quiz to help you be informed about your current knowledge of paediatric medical traumatic stress (PMTS) and responsive trauma-informed care.  Your score will be available in 'My Progress' using the link at the top of this page.  It is mandatory to complete this quiz prior to continuing with the e-learning package. | N/A | N/A |
| 1^a^ | Almost every child who is hospitalised for injury or illness has at least one acute stress reaction. | X | X |
| 2^a^ | It is inevitable that most children and families who experience a serious illness or injury will go on to develop significant posttraumatic stress. | X | X |
| 3^a^ | Children who are more severely injured or ill have more serious acute stress reactions than those who are less severely injured or ill. | X | X |
| 4^a^ | Children who, at some point during a distressing event, believe that they might die are at greater risk for posttraumatic stress reactions. | X | X |
| 5^a^ | Children who rate their pain as severe have the same risk of posttraumatic stress as other children. | X | X |
| 6^a^ | Children and families with significant posttraumatic stress reactions usually show obvious signs of distress. | X | X |
| 7^a^ | Psychological and physical recovery after a serious illness or injury are largely independent of each other | X | X |
| 8^a^ | Only children older than the age of 6 are at risk of developing serious posttraumatic stress following illness or injury. | X | X |
| 9^a^ | The best way to prevent serious posttraumatic stress is through structured intervention programs - small changes in everyday care interactions are not enough. | X | X |
| 10^b^ | Which of the following is a sign or symptom that a health professional may be experiencing secondary traumatic stress?  Avoiding having a challenging conversation  Brief irritation towards a co-worker  Intrusive thoughts | X | X |
| 11^b^ | What are the four steps of CARE?  Create a space, Action, Reflect & help, Evaluate  Compassion, Ask, Reflect, Engage  Create a Space, Ask & assess, Reflect, Evaluate |  | X |

Abbreviation: N/A = not applicable

^a^ Choose one response from unsure, agree, disagree

^b^ Choose one response from those listed
