# Supplementary material for: A web-based educational intervention to implement trauma-informed care in a paediatric healthcare setting: protocol for a feasibility study using pre-post mixed methods design
Source: Pilot Feasibility Stud. 2020 Aug 19;6:118. doi: 10.1186/s40814-020-00636-8 (PMC7436985; doi:10.1186/s40814-020-00636-8)
Supplement: Supplementary file 2 — Additional file 2. Feedback questions. Description: Questions to assess confidence, satisfaction and beliefs post Responsive CARE intervention [file 40814_2020_636_MOESM2_ESM.doc]

| Number | Question |
| --- | --- |
|  | **Confidence** |
| 1^a^ | As a result of this self-directed course, I am confident to contribute to responsive trauma-informed decision making that may impact a person's recovery and treatment. |
| 2^a^ | As a result of this self-directed course, I have increased my knowledge of the benefits of responsive trauma-informed healthCARE for children, families, and staff. |
| 3^a^ | As a result of this self-directed course, I am more confident to contribute to a team-based responsive trauma-informed care framework within my clinical setting. |
| 4^a^ | As a result of this e-learning package, I am confident I have skills to reduce the risk of traumatising children/families during the provision of interventions that can be painful and frightening. |
| 5^a^ | As a result of this e-learning package, I have increased my knowledge of the importance of implementing regular self-care. |
| 6^a^ | As a result of this e-learning package, I have skills to prevent PMTS and to manage children with PMTS. |
| 7^a^ | As a result of this e-learning package, I am more confident to integrate my knowledge of trauma-based responses to inform my clinical decision-making with children, regardless of their age, cultural background, special education needs etc. |
| 8^a^ | As a result of this self-directed course, I am more confident to integrate my knowledge of trauma-informed responses throughout the continuum of care for children receiving hospital services (for example, inpatient admission, outpatient appointments). |
|  | **Satisfaction** |
| 9^a^ | How likely would you be to recommend this e-learning package to a colleague? |
|  | **Beliefs** |
| 10a^b^ | How important is it for you to integrate responsive trauma-informed healthCARE into your daily work? |
| 10b^b^ | How likely is it that you will integrate responsive trauma-informed healthCARE into your daily work? |
| 10c^b^ | How confident are you to integrate responsive trauma-informed healthCARE into your daily work? |

^a^ 5-point response scale from 1 (not at all) to 5 (a lot)

^b^ 10-point response scale from 1 (not at all) to 10 (extremely)
