# Supplementary material for: A web-based educational intervention to implement trauma-informed care in a paediatric healthcare setting: protocol for a feasibility study using pre-post mixed methods design
Source: Pilot Feasibility Stud. 2020 Aug 19;6:118. doi: 10.1186/s40814-020-00636-8 (PMC7436985; doi:10.1186/s40814-020-00636-8)
Supplement: Supplementary file 3 — Additional file 3:. Overview of Responsive CARE course content. Description: Additional information about the Responsive CARE course content [file 40814_2020_636_MOESM3_ESM.doc]

| Section | Summary of content |
| --- | --- |
| Overview | This e-learning package has been designed for Health Professionals who provide hospital-based interventions to children and their families. It covers the continuum of care for children up to 18 years. |
|  |  |
| Module sequence | The course and the content are designed to progressively enhance your knowledge of responsive trauma-informed care in a healthcare setting. Therefore, the e-learning course has been designed so that you will progress through four modules in sequence. Once you have completed a module, you are allowed to return to that module at any time. |
|  |  |
| Learning objectives | 1. Increased knowledge (for example, the importance of Reflective Practice as a means to build self-awareness around personal toll and need for self-care)  2. Improved clinical reasoning based on an understanding of the responsive  healthCARE framework (for example, confidently integrate knowledge of trauma-based responses to inform clinical decision making across lifespan, continuum of care and healthcare context (e.g. inpatient, outpatient, community, rural/remote)  3. New skills to use in your work with children and families (for example, how to reduce the risk of traumatising children/families during the process of providing interventions that can be painful and frightening)  4. Increased confidence to prevent PMTS and to support children with PMTS. |
|  |  |
| Learning outcomes | Following completion of this course you should be able to (for example)  • understand that you can do a lot, even when time is limited.  By applying the responsive trauma-informed healthcare framework in your daily practice, we anticipate the following benefits for patients and yourself (for example)  • improved treatment adherence |
|  |  |
| Introduction | Consent to research participation (Additional File 6)  Pre-course quiz (Additional File 6) |
|  |  |
| Module 1 to Module 5 | Refer to Table 1 for overview of content |
| Resources | Includes resources (such as links to screening tools, cultural resources) for each module, reflective practice videos and references) |
