# Supplementary material for: A web-based educational intervention to implement trauma-informed care in a paediatric healthcare setting: protocol for a feasibility study using pre-post mixed methods design
Source: Pilot Feasibility Stud. 2020 Aug 19;6:118. doi: 10.1186/s40814-020-00636-8 (PMC7436985; doi:10.1186/s40814-020-00636-8)

Additional File 4 Educational presentation for recruitment of health professional users of the Responsive CARE intervention

CARE

Responsive Trauma-Informed Healthcare

An introduction to an e-learning package designed for Health Professionals who provide hospital-based interventions

Project Team:
Dr Megan Simons, Dr Alexandra De Young, Dr Zephanie Tyack, Ms Gillian Montague
Professor Roy Kimble, Professor Justin Kenardy,

Centre For Children's Burns & Trauma Research
Queensland Government
The University Of Queensland Australia

Introducing Jonathon

CARE

Fact file:
- Medical Practitioner
- Manager, Medical Workforce and Education

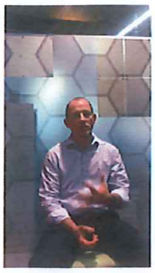

CARE

Course content

Bite size information grabs
Videos
Quizzes
Surveys
Clinical scenarios
Reflective Practice
Interactive Whiteboard Videos

CARE

Introduction: Consent to research + quiz

Module 1: Understanding Paediatric Medical Traumatic Stress (PMTS)

Module 2: Introducing the CARE framework

Module 3: How Health Professionals apply CARE

Module 4: Taking CARE of yourself

Conclusion: Feedback + quiz

Resources

---

4 STEPS OF CARE

CARE

C — **Create a space** that is suitable for the context you are in, and take a moment to STOP and focus on what is going on for the child, family member/s and yourself in that moment.

A — **Ask and assess** how the child and family member/s are coping

R — **Reflect** by observing what the child and family member/s are saying and doing, and implement a strategy to help if necessary

E — **Evaluate** if this interaction made a difference, and determine if anything else is needed

CARE

Introducing Daria

CARE

Fact file:
- Occupational Therapist
- Works in the Paediatric Persistent Pain Team, QCH

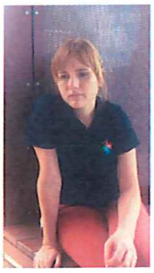

Course Home Content My Certificates My Progress Edit Course

(CHQ-M) Responsive Trauma-Informed HealthCARE

Welcome Gillian,

Available on https://ilearn.health.qld.gov.au/ with log-in details provided by project team
Email: Megan.Simons@health.qld.gov.au

CARE

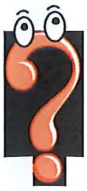

Supplement: Supplementary file 4 — Additional file 4. Introduction to Responsive CARE. Description: Slide content of introduction session for recruitment of users of the Responsive CARE intervention. [file 40814_2020_636_MOESM4_ESM.pdf]
