# Supplementary material for: A web-based educational intervention to implement trauma-informed care in a paediatric healthcare setting: protocol for a feasibility study using pre-post mixed methods design
Source: Pilot Feasibility Stud. 2020 Aug 19;6:118. doi: 10.1186/s40814-020-00636-8 (PMC7436985; doi:10.1186/s40814-020-00636-8)

Additional File 5 Educational information provided to participants about how to access the Responsive CARE intervention

**How to access the “Responsive trauma-informed healthcare e-learning package”.**

1. Go to iLearn: <https://ilearn.health.qld.gov.au/d2l/login>
2. Click on Back to iLearn MyHome


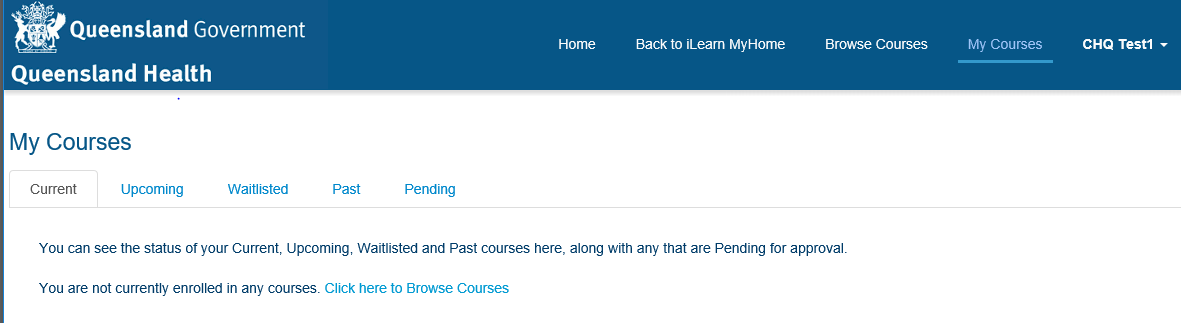


1. Enter your personal testor account details (**see a project team member to get details)


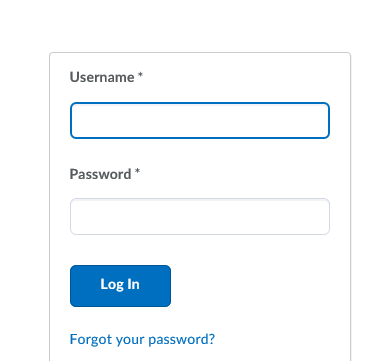


CHQ-Test

test2019

1. Navigate to the course.


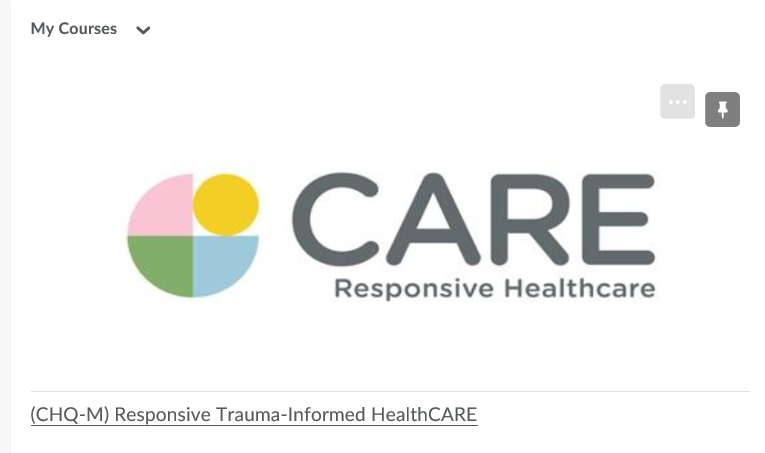


Click here

1. Open the course.


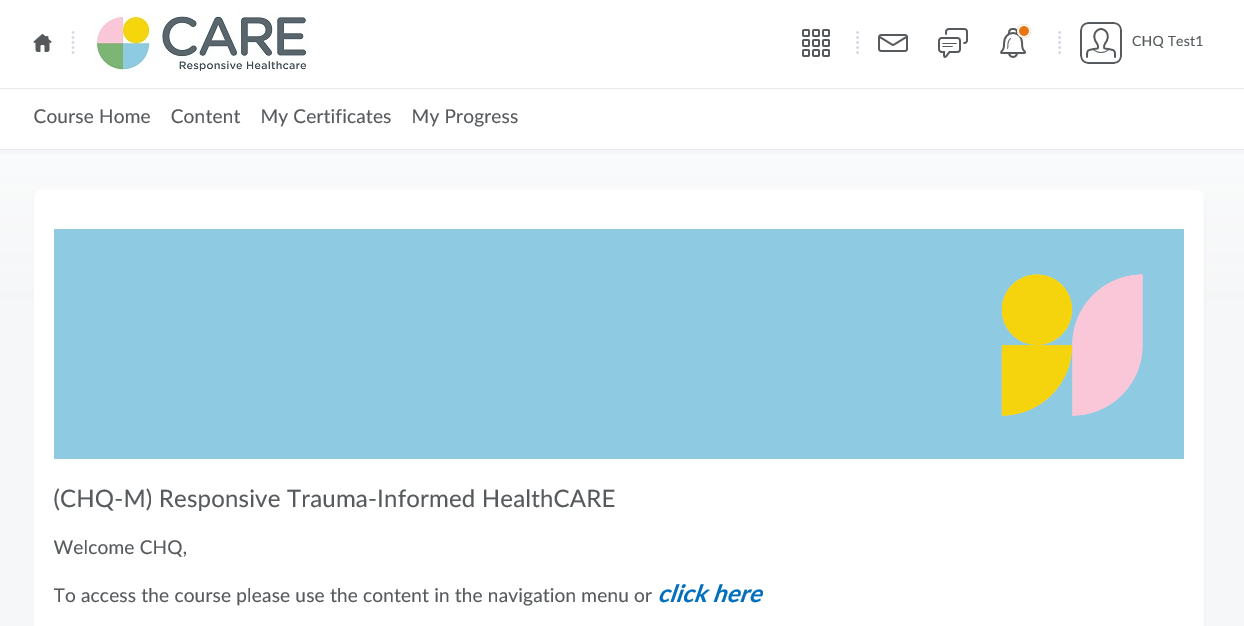


Click on content or click here to progress

1. If you are on a desktop or laptop, you will open to the overview page. If you are using a mobile, then you will open to the introduction module. Click on the introduction module to continue to progress through the course.


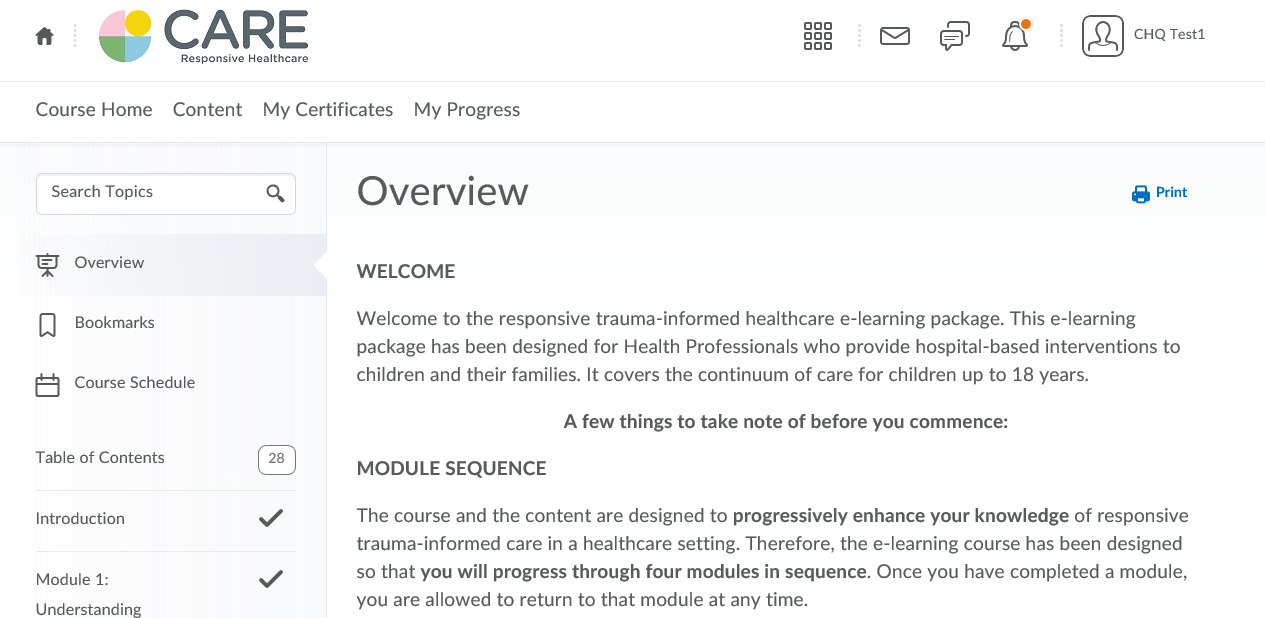


1. Click on ‘Consent to research participation’ to continue to progress through the course


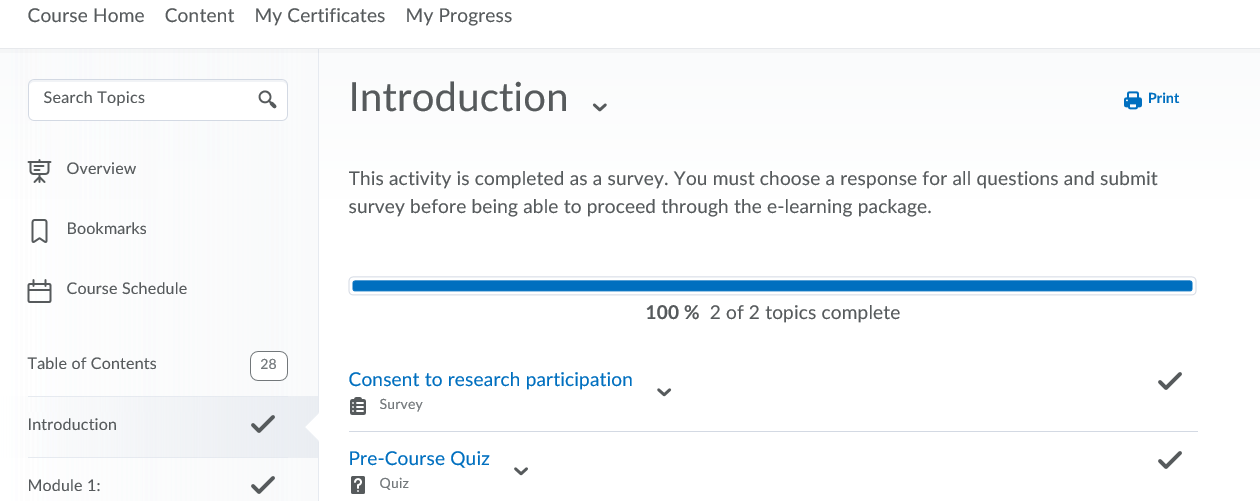


1. Use the arrows or navigation bar options to move through the course.


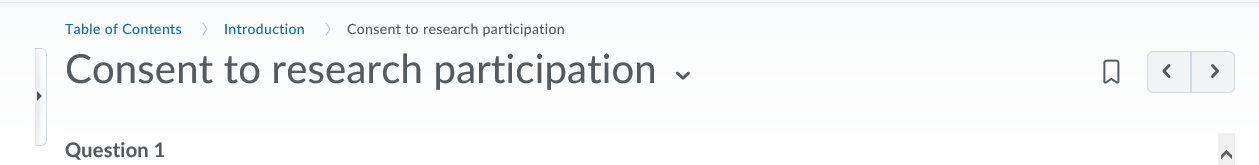

Supplement: Supplementary file 5 — Additional file 5. Educational material for staff to support access to Responsive CARE intervention [file 40814_2020_636_MOESM5_ESM.doc]
