# Supplementary material for: A web-based educational intervention to implement trauma-informed care in a paediatric healthcare setting: protocol for a feasibility study using pre-post mixed methods design
Source: Pilot Feasibility Stud. 2020 Aug 19;6:118. doi: 10.1186/s40814-020-00636-8 (PMC7436985; doi:10.1186/s40814-020-00636-8)
Supplement: Supplementary file 6 — Additional file 6. Demographic questions for Health Professionals. Description: Additional questions to describe health professionals completing Responsive CARE intervention [file 40814_2020_636_MOESM6_ESM.doc]

| Number | Question | Response |
| --- | --- | --- |
|  | **Consent to Research** |  |
| 1 | I acknowledge I have been provided with information regarding the research project and have signed a consent form. By marking the 'yes' box, I acknowledge that I am willing to have my responses and information that I provided when accessing the package used as part of a research project investigating responsive trauma-informed care practices in the Stuart Pegg Paediatric Burns Centre (Queensland Children's Hospital).  Yes  No | Choose one box only |
|  | **Demographics** |  |
| 2 | What is your profession?  Administration  Medical Practitioner  Music Therapist  Nurse  Occupational Therapist  Physiotherapist  Psychologist  Social Worker  Other | Choose one box only |
| 3 | How many years of experience have you had in hospital-based patient care (in total)? | Free text response |
| 4 | How many of those years were in your current role in burns care? | Free text response |
| 5 | What is your gender?  Male  Female  Other | Choose one box only |
| 6 | What is your age? | Free text response |
| 7 | How many hours of specific training in trauma-informed (or psychosocial) care have you had?  No training  1 to 10 hours of training  11 to 20 hours of training  21 to 40 hours of training  More than 40 hours of training | Choose one box only |
| 8 | How long ago did you complete the specific training in trauma-informed (or psychosocial) care?  I have not completed specific training  Within the past year  1 to 4 years ago  5 to 10 years ago  More than 10 years ago | Choose one box only |
